# Posttraumatic Refractory Intracranial Hypertension and Brain Herniation Syndrome: Cerebral Hemodynamic Assessment before Decompressive Craniectomy

**DOI:** 10.1155/2013/750809

**Published:** 2013-11-27

**Authors:** Edson Bor-Seng-Shu, Wellingson Silva Paiva, Eberval G. Figueiredo, Yasunori Fujimoto, Almir Ferreira de Andrade, Erich Talamoni Fonoff, Manoel Jacobsen Teixeira

**Affiliations:** ^1^Division of Neurological Surgery, Hospital das Clinicas, University of Sao Paulo, School of Medicine, 255 Eneas Aguiar Street, Office 4079, 05403010 Sao Paulo, SP, Brazil; ^2^Department of Neurosurgery, Osaka University Graduate School of Medicine, Osaka, Japan

## Abstract

*Background*. The pathophysiology of traumatic brain swelling remains little understood. An improved understanding of intracranial circulatory process related to brain herniation may have treatment implications. *Objective*. To investigate the cerebral hemodynamic changes associated with brain herniation syndrome due to traumatic brain swelling. *Methods*. Nineteen head-injured patients with evidence of refractory intracranial hypertension and transtentorial herniation were prospectively studied. Cerebral hemodynamic assessment by transcranial Doppler (TCD) ultrasonography was performed prior to decompressive craniectomy. Patients and their cerebral hemispheres were classified according to TCD-hemodynamic patterns, and the data correlated with neurological status, midline shift on CT scan, and Glasgow outcome scale scores at 6 months after injury. *Results*. A wide variety of cerebral hemodynamic findings were observed. Ten patients (52.7%) presented with cerebral oligoemia, 3 patients (15.8%) with cerebral hyperemia, and 6 patients with nonspecific circulatory pattern. Circulatory disturbances were more frequently found in the side of maximal cerebral swelling than in the opposite side. Pulsatility index (PI) values suggested that ICP varied from acceptable to considerably high; patients with increased PI, indicating higher microvascular resistance. No correlation was found between cerebral hemodynamic findings and outcome. *Conclusions*. There is a marked heterogeneity of cerebral hemodynamic disturbances among patients with brain herniation syndrome.

## 1. Introduction

The pathophysiology of traumatic brain swelling remains little understood to date; as a consequence, there are few therapeutic options to control this condition and a number of patients develop increased intracranial pressure (ICP), brain ischemia, and herniation [1–23].

To our knowledge, there are no studies concerning disturbances of intracranial circulation associated with the process of brain herniation and refractory intracranial hypertension due to severe traumatic brain swelling. An improved understanding of intracranial circulatory process related to this condition may have treatment implications.

The objective of this study was to investigate the cerebral hemodynamic changes associated with refractory elevated ICP and brain herniation syndrome due to traumatic brain swelling. The relationships between such cerebral hemodynamic patterns and the patients' outcome, as well as between cerebral hemodynamic patterns and computerized tomography (CT) scan findings, were also verified.

## 2. Clinical Material and Methods

### 2.1. Study Design and Patient Enrollment

Traumatic brain injury (TBI) patients with clinical and tomographic signs of intracranial hypertension and brain herniation due to uncontrollable brain swelling for whom decompressive craniectomy was indicated and in whom transcranial Doppler (TCD) ultrasonography had been performed were prospectively enrolled. Exclusion criteria were penetrating head injuries, Glasgow Coma Scale (GCS) score of 3 associated with bilaterally fixed and dilated pupils, and impossibility to assess cerebral circulation by TCD ultrasonography. Patients with multisystem trauma were not excluded. Demographic, clinical, and radiological data were collected for every patient. This study was approved by our research ethics committee and conducted according to the Declaration of Helsinki principles.

### 2.2. Management Protocol

All patients were managed by a standard regimen based on the guidelines of the American College of Surgeons (Advanced Trauma Life Support) and of the American Association of Neurological Surgeons as described in our previous report [4]. After stabilization of respiratory and circulatory functions, patients with neurological deterioration and clinical signs of brain herniation and raised ICP underwent urgent computerized tomography (CT) scans of the brain. Routine ICP monitoring was not intended in this protocol.

### 2.3. Clinical and Tomographic Definitions of Brain Herniation

Clinical evidence of cerebral herniation syndrome consisted of neurological deterioration characterized by decrease in GCS score and/or dilation of pupils that were unresponsive to light, unilaterally or bilaterally, in conjunction with CT evidence of brain herniation characterized by severe diffuse brain swelling or predominantly unilateral diffuse brain swelling associated with mass effect, a shift of midline cerebral structures >5 mm, and/or obliteration of perimesencephalic cisterns. Patients with persisting GCS score of 3 and/or bilaterally fixed and dilated pupils did not undergo decompressive craniectomy and were excluded.

### 2.4. Assessment Technique of Cerebral Hemodynamics

The neurosurgical team was oriented to maintain their routine practice, without waiting for the arrival of TCD physician to avoid any delay in the patients' treatment. In most instances, TCD measurements were obtained, while the patient waited to go into the operating theater (one case) or while the anesthesiologist prepared the patient in the operating room.

The middle cerebral artery (MCA) and the distal segment of the extracranial internal carotid artery (ICA) were insonated using a portable 2 MHz pulsed TCD device (Pioneer TC 2020 EME; Nicolet Biomedical, Madison, WI) via temporal and submandibular ultrasound windows, respectively. The MCA and extracranial ICA blood flow variables that were recorded and analyzed included the mean velocity (the time mean of the peak velocities over the course of four cardiac cycles) and the pulsatility index (PI = [systolic velocity – diastolic velocity]/mean velocity).

Physiological data, including hematocrit, arterial blood carbon dioxide and oxygen pressures, body temperature, and systemic arterial blood pressure, were documented in each TCD study.

### 2.5. Definition of  TCD Hemodynamic Patterns

Elevated TCD flow velocities have been found in both cerebral vasospasm and hyperemia. Given that the extracranial ICA is not involved in the spasm process, flow velocity changes within this artery are associated with changes in CBF rather than with changes in arterial diameter [24–27]. Extracranial ICA flow velocity can be considered as an index of CBF since elevated flow velocity in the extracranial ICA is associated with increased flow volume to the brain [24–28]. Lindegaard ratio (LR), defined as the ratio of MCA mean flow velocity to extracranial ICA mean flow velocity, can differentiate cerebral vasospasm from hyperemia [25]. In hyperemia, both extracranial ICA and MCA mean flow velocities are increased, and the LR remains <3. In the present study, MCA mean flow velocities >100 cm/second in conjunction with LR <3 were defined as indicative of cerebral hyperemia, while MCA mean flow velocities <40 cm/second were defined as indicative of cerebral oligoemia. MCA mean flow velocities between 40 and 100 cm/second along with LR <3 were considered as nonspecific hemodynamic pattern.

### 2.6. Categorization of Patients by Cerebral Hemodynamic Patterns

Patients with hyperemia in both cerebral hemispheres or hyperemia in one cerebral hemisphere and nonspecific circulatory pattern in another hemisphere were considered as presenting cerebral hyperemia. On the other hand, patients with oligoemia in both cerebral hemispheres or oligoemia in one cerebral hemisphere and nonspecific hemodynamic pattern in the opposite hemisphere were defined as having cerebral oligoemia. Patients with hyperemia in one cerebral hemisphere and oligoemia in the contralateral hemisphere were grouped separately.

### 2.7. Data Collection

Information on age, gender, date of accident, time intervals between the accident and hospital admission and between admission and surgical treatment, mechanism of injury, neurological status (GCS score and pupil response) at admission, before, and after decompressive craniectomy, shift degree of midline cerebral structures, associated intracranial lesions, length of hospital stay, and outcome were registered. Outcome was evaluated at 6 months after injury by Glasgow Outcome Scale (GOS) score. Patients with good recovery (GOS score of 5) or with moderate disability (GOS score of 4) were considered as presenting favorable outcome, while patients with severe disability (GOS score of 3) or in a vegetative state (GOS score of 2) or those who died (GOS score of 1) were defined as having unfavorable outcome.

### 2.8. Statistical Analysis

Data were reported as means ± standard deviations. The Mann-Whitney *U* test, the Wilcoxon rank-sum test, and the Fischer exact test were performed. Spearman correlation coefficients were calculated when appropriate. The tests were considered statistically significant at a probability value <0.05.

## 3. Results

### 3.1. Patient Characteristics

Nineteen patients with severe traumatic brain swelling and evidence of elevated ICP and transtentorial herniation were included. The mean age of the patients was 33 ± 14 years (range 17–63 years). There were 13 males and 6 females. Admission GCS scores ranged from 4 to 13, with a median of 7. During TCD examinations, GCS scores ranged from 4 to 12 (median 6). Twenty percent of patients had a hypotensive insult at hospital admission. All patients had clinical and tomographic signs of cerebral herniation. Two patient subgroups were formed. The first group included 9 patients without focal lesions, in whom severe traumatic brain swelling accompanied by therapy-resistant signs of brain herniation required decompressive craniectomy. The second group included 10 patients who had a space-occupying hematoma (contusion hemorrhage and extradural or subdural hematoma) that had been removed in an initial operation, and developed afterwards severe brain swelling concomitant with signs of transtentorial herniation. Demographic, clinical, and imaging features for each patient were summarized in Table 1.

### 3.2. CBF Velocity Measurements and PI

Mean flow velocities in the MCA ranged widely from 8 to 143 cm/s. The average MCA mean flow velocities were 53 ± 38 cm/s and 51 ± 26 cm/s, respectively, in the most swollen cerebral hemisphere and in the contralateral hemisphere. The MCA PI varied from 0.61 to 7.09; the average MCA PI was 1.85 ± 1.56 in the most swollen hemisphere and 1.73, contralaterally. All patients with MCA mean flow velocity <40 cm/s presented PI >1.00, and those with MCA mean flow velocity >100 cm/s presented PI <0.63. The LR was <3 in all cases.

### 3.3. Classification of Patients and Their Cerebral Hemispheres by Hemodynamic Patterns

Ten patients (52.7%) were considered as having cerebral oligoemia, 3 patients (15.8%) met the criteria for cerebral hyperemia, and 6 patients (31.5%) were found to present nonspecific circulatory pattern. No patients were found to have hyperemia in one cerebral hemisphere and oligoemia in the opposite hemisphere (Table 2).

In the most swollen cerebral hemisphere, 52.6% of the patients presented MCA mean flow velocities <40 cm/s (cerebral oligoemia), 36.9% between 40 and 100 cm/s (nonspecific hemodynamic pattern), and 10.5% >100 cm/s (cerebral hyperemia). In the opposite side, 17.6% of the patients had MCA mean flow velocities <40 cm/s (cerebral oligoemia), 76.5% between 40 and 100 cm/s (nonspecific hemodynamic pattern), and 5.9% >100 cm/s (cerebral hyperemia) (Table 2). Abnormal hemodynamic patterns were more frequently found in the most swollen cerebral hemisphere than in the opposite hemisphere (63.1% versus 23.5%, resp.) (*P* < 0.05).

### 3.4. Cerebral Hemodynamic Patterns and Outcome

No correlation was found between cerebral hemodynamic patterns and other variables such as preoperative GCS score, the degree of midline cerebral structures shift on preoperative CT scan, GOS scores at 6 months after injury, and neurological recovery according to favorable (good recovery and moderate disability) or unfavorable outcome (severe disability, vegetative state, or death) at 6 months followup.

## 4. Discussion

### 4.1. Overview of Results

**Figure 1:**
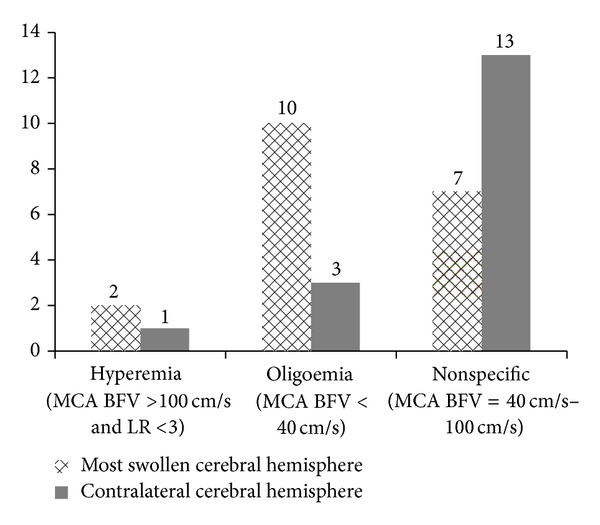
Hemodynamic patterns in the most swollen cerebral hemisphere and in the contralateral hemisphere (number of patients versus hemodynamic pattern in TCD). LR: Lindegaard ratio; MCA: middle cerebral artery; BFV: blood flow velocity; TCD: transcranial Doppler ultrasonography; All patients presented LR <3.

We report a series of 19 patients with traumatic brain swelling and evidence of increased ICP and brain herniation, refractory to clinical measures, in whom cerebral hemodynamic assessment by TCD ultrasonography was performed prior to decompressive craniectomy. Participants and their cerebral hemispheres (the side of maximal cerebral swelling and the opposite side) were categorized according to TCD hemodynamic patterns, and the findings correlated with preoperative GCS score, degree of midline cerebral structures shift on preoperative CT scan, GOS scores at 6 months after injury, and 6-month follow-up neurological outcome based on favorable or unfavorable recovery. To our knowledge, this is the first study that systematically evaluated the cerebral blood circulation of patients with traumatic brain swelling leading to uncontrollable raised ICP and brain herniation syndrome. Patients with severe brainstem injury (GCS score of 3 and/or bilaterally fixed and dilated pupils) were excluded from this study; therefore, this investigation refers to patients eligible for treatment. We showed a wide diversity of cerebral hemodynamic findings, ranging from severe oligoemia to hyperemia. Ten patients (52.7%) presented with cerebral oligoemia, 3 patients (15.8%) with cerebral hyperemia, and 6 patients (31.5%) with nonspecific circulatory pattern (Table 2). Hemodynamic abnormalities were more frequently found in the side of maximal cerebral swelling than in the opposite side (63% versus 23%, resp.) (Figure 1). PI values suggested that ICP varied from acceptable to considerably high; patients with increased PI, indicating high microvascular resistance, tended to have cerebral oligoemia, whereas those with decreased PI, reflecting low microvascular resistance, tended to have cerebral hyperemia. No correlation was found between preoperative cerebral hemodynamic patterns and GOS scores at 6 months after injury and neurological recovery according to favorable (good recovery and moderate disability) or unfavorable outcome (severe disability, vegetative state, or death) at 6 months followup.

### 4.2. Methodological Issues

A potential criticism of our study is the absence of ICP measurements, quantitative estimations of CBF, cerebral arteriovenous oxygen difference, cerebral metabolic rate of oxygen consumption, and biochemical markers for cerebral ischemia. However, in our department, we have used clinical monitoring, measurements by noninvasive methods, and serial imaging. We have used ICP monitoring only in selected cases with findings suggestive of intracranial hypertension in the neurosonograhic assessment.

The management of severe TBI without ICP monitoring in all patients has been confirmed in the recent study by Chesnut et al. [24], presenting the benefit of ICP monitoring for the outcome in randomized prospective trial. Measuring of the other physiological variables also requires the use of invasive cerebral and jugular venous catheters, as well as time-consuming procedures that are difficult to justify in patients with neurological deterioration and evidence of uncontrollable intracranial hypertension and brain herniation. TCD study cannot provide quantitative blood flow data, such as flow rate, expressed in mL/min, or tissue perfusion, expressed in mL/100 g of brain tissue/min [3, 4]; however, blood flow velocities in the cerebral arteries allow detection of cerebral hyperemia, oligoemia, and vasospasm [25–28]. Despite the limitations of TCD ultrasonography, its noninvasiveness and the possibility of rapid real-time acquisition of intracranial hemodynamic data made this study possible.

For interpreting the results of TCD ultrasonography, it is necessary to consider the possibility that both cerebral hyperemia (elevated CBF) and vasospasm usually occur more noticeably as of 2 days after injury [12, 28]. Blood flow velocities >100  cm/second should be interpreted with caution given that high blood flow velocities in the cerebral arteries can be found in hyperemia as well as in vasospasm [25–27]; LR was used in our study to differentiate hyperemia from vasospasm [26].

Our results failed to demonstrate a correlation between cerebral hemodynamic patterns and GOS scores at 6 months after injury and between cerebral hemodynamic patterns and neurological recovery according to favorable or unfavorable outcomes at 6 months followup, although this fact does not mean that those correlations cannot exist because of the limitations of this investigation which include small patient sample, heterogeneous characteristics of the patient population, coexistence of several prognostic factors, and the complexity of the brain's hemimetabolic phenomena.

### 4.3. Previous Investigations

In the Morgalla et al.'s series comprising 33 head-injured patients who underwent decompressive craniectomy, preoperative TCD ultrasonography disclosed high resistance CBF pattern in 24% of patients, systolic spikes in 12% of patients, systolic flow pattern with absent diastolic flow in 57% of patients, and normal TCD flow pattern in 12% of patients [29]. These data indicate occurrence of cerebral oligoemia and nonspecific circulatory pattern in the setting of refractory raised ICP and brain herniation, which are quite intuitive and understandable. Although the characterization of TCD flow velocities was not the purpose of the study and there was no description of cerebral hyperemia in this series [29], these data also revealed a wide variability in cerebral hemodynamic presentations which are in line with our findings. On the other hand, it is reasonable to accept the occurrence of cerebral hyperemia in patients with uncontrollable increased ICP and brain herniation, as seen in our series. Kelly et al. [30, 31] performed a physiological stratification of TBI patients based on CBF and ICP measurements and described five subgroups of patients, each exhibiting relatively unique clinical courses and outcomes: (1) hyperemia without intracranial hypertension, (2) hyperemia with intracranial hypertension and favorable outcome, (3) cerebral vasoparalysis and hyperemia with intracranial hypertension and poor outcome (our patients), (4) intracranial hypertension without hyperemia (our patients), and (5) normal ICP without hyperemia.

Interestingly, Morgalla et al. [29, 32] have considered TCD studies as an important adjunct in decision making, monitoring cerebral hemodynamic deterioration to systolic flow pattern (with absent diastolic flow) in some cases. In our series, TCD data were not a deciding factor for surgical decompression, which was indicated based on clinical and CT findings compatible with raised ICP and brain herniation. We were afraid of waiting for the cerebral diastolic flow to cease since at this time CPP is certainly quite impaired and the indication of surgical treatment may be too late for some patients [4]. Another option to measure hemodynamic changes in severe TBI patients is contrast-enhanced ultrasonography (CEU). Heppner et al. [33] have used this technique for noninvasive evaluation of cerebral perfusion in patients with traumatic brain injury and to assess the effect of decompressive surgery on cerebral perfusion as measured by CEU. The authors studied with TBI undergoing decompressive craniectomy and concluded that this ultrasonography method has potential for the intraoperative and bedside assessment of cerebral perfusion in patients with TBI.

### 4.4. Implications for Treatment

Current therapeutic strategy concerning CBF management in head-injured patients is to avoid states of critical cerebral hyperemia and oligoemia [4, 34, 35]. In theory, the former can lead to further increase in cerebral blood volume, vasogenic edema, and the risk of intracerebral bleeding, while the latter can induce cerebral ischemia and infarction. Both states can worsen brain swelling and intracranial hypertension. For these reasons, decompressive craniectomy as the sole treatment could be insufficient in these cases [4, 36]. Efforts must be made to maintain adequate cerebral hemodynamics, preferably coupled with metabolism, avoiding significant cerebral hyperemia and oligoemia. Factors that can aggravate cerebral hyperemia such as hypercapnia, anemia, arterial blood hypertension, increased cardiac output, hypervolemia, and cerebral metabolic crisis, among others, must be investigated and corrected if possible. In contrast, factors that can intensify cerebral oligoemia, such as hypocapnia, arterial blood hypotension, decreased cardiac output, hypovolemia, dehydration, and raised ICP must be ruled out and treated when appropriate. Factors that increase cerebral metabolic activity, such as hyperthermia and seizures, must be kept in mind, irrespective of cerebral hemodynamic pattern, whether hyperemia or oligoemia. Despite not being demonstrated in our series, some patients can present with hemodynamic pattern suggestive of cerebral hyperemia in one hemisphere and oligoemia in the other [35]. Treatment of these patients, mainly if both hyperemia and oligoemia are critically severe, may be challenging. Triple-H therapy or surgical decompression indicated for treating cerebral oligoemia is clearly not suitable for the hemisphere with hyperemia. Such patients should be monitored closely with multimodal fashion to achieve a middle ground whereby correction of cerebral hypoperfusion does not cause significant worsening of contralateral cerebral hyperemia [35]. For the future, in addition to CBF and ICP management, treatment at the biochemical level can be devised to manage brain swelling associated with posttraumatic cerebral metabolic crisis [36, 37]. Although there is no evidence to support the routine use of surgical decompression to reduce unfavorable outcomes in severe TBI and refractory high ICP, decompressive craniectomy has been the treatment of choice [1–4, 6–9, 11–15, 17–23, 29, 32, 38, 39].

## 5. Conclusions

There is a considerable heterogeneity of cerebral hemodynamic findings among individuals with refractory intracranial hypertension and brain herniation syndrome due to traumatic brain swelling, and also between their cerebral hemispheres. These findings are in accordance with previous publications on TBI, which demonstrated cerebral heterogeneity in terms of circulation, pressure autoregulation, critical closing pressure, oxygenation, and metabolism. This supports the concept of heterogeneous nature of the pathophysiology of the TBI and of the patients' outcomes, suggesting that therapeutic measures should be planned individually. The recognition of posttraumatic cerebral hemodynamic patterns and their significances is potentially useful for devising more specific therapeutic strategies.

## Figures and Tables

**Table 1:** Summary of patients' demographic, clinical, and radiological data*.

Case no.	Age (yrs), Sex	Lesions operated at hospital admission	Interval from admission to TCD	GCS score	Pupils	Midbrain cisterns	Midline shift on CT (mm)	Side of maximal cerebral swelling	CHP	6-Mo GOS score
1	63, F		6 hours	6	Unequal	Absent	12	Right	Oligoemia	1
2	18, M		4 hours	6	Unequal	Absent	16	Right	Nonspecific	1
3	28, M		6 days	6	Unequal	Absent	9	Left	Oligoemia	5
4	39, F		7 hours	6	Unequal	Absent	11	Right	Hyperemia	3
5	28, M		3 hours	7	Unequal	Absent	15	Right	Oligoemia	4
6	25, M		4 days	6	Unequal	Absent	12	Right	Oligoemia	3
7	19, F	ICH	24 hours	6	Unequal			Right	Nonspecific	4
8	28, F		2 hours	6	Unreactive	Absent	9	Left	Oligoemia	4
9	22, M		2 hours	6	Unequal	Absent	14	Right	Hyperemia	2
10	25, F		6 days	5	Unequal	Absent	8	Right	Oligoemia	1
11	30, M	ICH	2 days	6	Unequal	Absent	17	Right	Oligoemia	1
12	27, M	SDH	8 days	6	Unequal	Absent	12	Right	Nonspecific	5
13	17, M	SDH	3 days	10	Unequal	Absent	5	Left	Hyperemia	4
14	39, M	EDH	3 days	12	Unequal			Right	Oligoemia	4
15	61, F	ICH SDH	3 days	9	Unequal	Absent	11	Left	Oligoemia	2
16	51, M	ICH	4 days	9	Unequal	Absent	19	Left	Nonspecific	3
17	43, M	SDH	3 days	6	Unequal	Absent	20	Left	Nonspecific	1
18	46, M	SDH	9 days	4	Unequal	Absent	10	Right	Oligoemia	2
19	23, M	ICH	1 day	6	Unreactive	Absent	15	Left	Nonspecific	3

*EDH: extradural hematoma; GCS: Glasgow Coma Scale; GOS: Glasgow Outcome Scale; ICH: traumatic contusional intracerebral hemorrhage; SDH: subdural hematoma; Unequal: unequal, at least one reactive; Unreactive: unreactive bilaterally; CHP: cerebral hemodynamic patterns.

**Table 2:** Number of patients according to cerebral hemodynamic patterns.

Hemodynamic patterns	No. of patients
Hyperemia	3 (15.8%)
Oligoemia	10 (52.7%)
Nonspecific	6 (31.5%)
